# Comparative Efficacy of Dexlansoprazole, Pantoprazole, Esomeprazole, and Rabeprazole in Achieving Optimal 24-Hour Intragastric pH Control: A Randomized Crossover Study Using Ambulatory pH Monitoring

**DOI:** 10.7759/cureus.71418

**Published:** 2024-10-14

**Authors:** Umesh Jalihal, Jyoti R Mahapatra, Ajit Kumar, Tarun Bharadwaj, Harsh D Singh, Vatsal Mehta, Dinesh R Patil, Onkar C Swami

**Affiliations:** 1 Gastroenterology, Karnataka Gastro Centre, Bengaluru, IND; 2 Gastroenterology, Peerless Hospital, Kolkata, IND; 3 Gastroenterology, KIMS Hospital, Hyderabad, IND; 4 Gastroenterology, ApolloSAGE Hospital, Bhopal, IND; 5 Gastroenterology, Sukhbir Hospital, Amritsar, IND; 6 Gastroenterology, Ivy Hospital, Amritsar, IND; 7 Gastroenterology, Health1 Super Speciality Hospital, Ahmedabad, IND; 8 Clinical Pharmacology, Alembic Pharmaceuticals Ltd, Mumbai, IND; 9 Gastroenterology and Clinical Pharmacology, Alembic Pharmaceuticals Ltd, Mumbai, IND

**Keywords:** dexlansoprazole, esomeprazole, intragastric ph, pantoprazole, proton pump inhibitors, rabeprazole

## Abstract

Introduction: Proton pump inhibitors (PPIs) regulate gastric acid reflux. Dexlansoprazole's efficacy in prolonging acid suppression compared to conventional PPIs and placebo requires evaluation.

Methods: A prospective, randomized, placebo-controlled, five-way crossover pilot study was conducted on healthy volunteers comparing the potency of dexlansoprazole to conventional PPIs in which five patients were randomized into five treatment cohorts, including dexlansoprazole 60 mg, pantoprazole 40 mg, esomeprazole 40 mg, rabeprazole 20 mg, and placebo, assessing 24-hour intragastric pH using Z/pH Recorder (ZepHr®, Diversatek, Inc., Milwaukee, WI) and analyzing statistical differences via paired t-test.

Results: Dexlansoprazole showed significantly longer durations with pH > 4.0 compared to placebo (P < 0.001) and all other PPIs (P < 0.05) over 24 hours. Although not significant in the first 0-12-hour period, dexlansoprazole maintained significantly higher pH levels in the last 12-24-hour period compared to pantoprazole (P = 0.001) and esomeprazole (P = 0.044) but not with rabeprazole (P = 0.075). Additionally, during the 24-hour pH monitoring measured at 30-minute intervals, dexlansoprazole (mean pH = 3.98 ± 0.11) consistently showed higher values than pantoprazole (mean pH = 3.48 ± 0.12), rabeprazole (mean pH = 3.66 ± 0.05), esomeprazole (mean pH = 3.66 ± 0.05), and placebo (mean pH = 2.52 ± 0.12), indicating its superior potency.

Conclusion: Dexlansoprazole's dual-delayed release mechanism demonstrates superior acid suppression compared to traditional PPIs and placebo in this pilot study. Larger studies are needed to further evaluate its long-term efficacy and safety.

## Introduction

Gastroesophageal reflux disease (GERD) is characterized by the reflux of abnormal stomach contents into the esophagus [1,2]. GERD symptoms are observed within 15-20% of adults residing in the United States; on the other hand, its prevalence ranges from 6.3% to 18.3% in Asian nations with roughly around 7.6-30% in the Indian population [3-7]. GERD encompasses erosive esophagitis (EO) and non-erosive reflux disease (NERD), with NERD accounting for the majority (70%) of GERD instances [8].

The treatment of GERD involves the use of a variety of methods, including lifestyle modifications, medication, and surgery [9-11]. As of now, the mainstay of GERD therapy includes medications, specifically proton pump inhibitors (PPIs) [12,13]. They comprise omeprazole 20 mg, lansoprazole 30 mg, rabeprazole 20 mg, esomeprazole 40 mg, pantoprazole 40 mg, and dexlansoprazole 60 mg and 20 mg, all of which have been authorized by the Food and Drugs Administration (FDA) for a duration ranging from four to eight weeks for GERD patients [14-17].

The mechanism of action of PPIs includes irreversible repression of the H+K+ATPase enzyme, which is involved in the terminal stage of gastric acid biosynthesis ultimately culminating in the reduced production of gastric acid within the stomach allowing for a lesser backflow into the esophagus [18,19]. A suitable amount of medication must be maintained during proton pump activation for PPIs to achieve optimal effectiveness in the stomach [18,20,21]. About 25% of the proton pump remains continuously operational, which undergoes a process of renewal every day [22]. Hence, when administered in a once-daily manner, PPIs owing to their short half-lives, exhibit reduced concentration in the latter part of the 24-hour dosing interval. For instance, rabeprazole, esomeprazole, and pantoprazole have half-lives of ~1.2 hours, therefore the acid suppression effects last only about 12-15 hours of the day. This allows for the reactivation of acid secretion by newly formed proton pumps leading to a sub-optimal therapeutic response of these PPIs during the latter part of 24-hour dosing [21,23,24]. As a result, conventional once-daily dosing fails to achieve sustained regulation of gastric acid secretion throughout the 24-hour cycle [24].

The recently developed dexlansoprazole dual modified release (MR) formulation is an advancement of conventional PPIs formulated from the R-enantiomer of lansoprazole and designed to address GERD and its associated symptoms. In contrast to other PPIs, dexlansoprazole is available in capsule form with a dual delayed-release mechanism [25,26]. Dexlansoprazole possesses a distinctive capability to release its active ingredient in both the duodenum and the small intestine, rendering it a dual-release agent [27]. After consumption, dexlansoprazole is released twice: first within one to two hours and again four to five hours later [28]. As a consequence of this, two and five hours after delivery, two peak concentrations are achieved at different periods respectively [29,30]. Dexlansoprazole MR ensures the most extensive preservation of drug concentration within the plasma and the lengthiest duration of proton pump inhibitory effect compared to all approved PPIs [30].

According to our extensive review of the literature, there is currently no comprehensive Indian dataset available that compares various PPIs within a single study setup. The present study was a prospective, randomized, placebo-controlled, five-treatment, five-phase, five-way crossover, pilot, investigator-initiated academic study to compare the pharmacodynamic effects of four different PPIs (i.e., dexlansoprazole, pantoprazole, esomeprazole, and rabeprazole) in healthy adult subjects. The primary objective of the study was to compare the efficacy of dexlansoprazole, pantoprazole, esomeprazole, and rabeprazole in achieving optimal intragastric pH suppression over 24 hours in healthy subjects. A placebo group was also included in the group to provide a control group, justifying an unbiased comparison of PPI's efficacy, and to help distinguish PPI's true pharmacological effect from probable psychological factors (placebo effect) that might impact clinical outcomes.

## Materials and methods

Study design

This was a prospective, randomized, placebo-controlled, five-treatment, five-phase, five-way, crossover, pilot, investigator-initiated study.

Baseline characteristics

Men and women aged 20-35 years who displayed normal health as established by the screening process by screening inclusion and exclusion criteria pertaining to clinical examination and individual medical history, those willing to follow the protocol, and those who provided consent to participate were eligible for enrolment.

The exclusion criteria were pregnant or lactating females; patients having a history of gastritis, acidity, gastric ulcer, anaphylaxis, or hypersensitivity reaction to benzimidazoles, dexlansoprazole, pantoprazole, esomeprazole, rabeprazole, or any other PPIs to prevent any related adverse events; patients who had used any medication/vaccine/medical intervention in the last two weeks prior to screening or with any underlying comorbidity, any history of significant alcoholism or drug abuse within the past one-year, significant smoking (more than 10 cigarettes per day) or consumption of tobacco product; subjects with any other clinically significant disorder or any other reason for which the investigator felt that the subject should not participate. Withdrawal criteria included intercurrent illness, protocol violations, unacceptable concomitant medication, and the investigator's discretion.

Treatment

The study groups consisted of the following: test (dexlansoprazole 60 mg), reference 1 (pantoprazole 40 mg), reference 2 (esomeprazole 40 mg), reference 3 (rabeprazole 20 mg), and reference 4 (placebo). All subjects participating in the study received a single dose of a PPI in each period according to the computer-generated randomization schedule. The placebo included an empty capsule shell of a B-complex. Dispensation of drugs was carried out by the research team out of sight of the subject and investigator. While concomitant therapy with medications unrelated to the study medication may have been allowed as deemed necessary by the investigator, no medication was permitted at least 48 hours prior to the scheduled dosing.

The study was conducted from October 2023 to December 2023. It was carried out for a duration of 20-21 days with a washout period of at least five days of each dosing to ensure accurate results and avoid any carryover effect of the treated drugs. During each dosing day, subjects reported to the hospital at 07:30 hours after an overnight fast of at least 10 hours.

Outcome measure

The intragastric pH was measured using a Z/pH Recorder (ZepHr®, Diversatek, Inc., Milwaukee, WI). The pH electrode was calibrated before and after each recording using standard buffer solutions of pH 1.68 and pH 7.00. The intragastric pH electrode was inserted and placed in the gastric antrum about 5 cm below the gastroesophageal junction via the intranasal route after topical application of local anesthetic gel (xylocaine 2%), with 24-hour ambulatory pH measurements commencing at 08:00 hours and ending at 08:00 hours the following day. Each subject received a single dose of the study medication at 8:30 hours with 200 ml of water. Post-dosing, each subject remained housed in the hospital under close observation until the next day to complete the whole 24-hour time period required for the study. Subjects were provided standardized meals at specific times of the day (10:30 hours, 13:30 hours, and 19:30 hours), with water available ad libitum throughout the stay. Other beverages, smoking, and tobacco chewing were strictly prohibited to avoid interference with the study results. Each subject’s vital signs, pH recordings, and general health were observed continuously for at least 24 hours during each period to ensure adherence to the study protocol.

Assessment of efficacy focused on intragastric pH measurements. The primary endpoint was the mean percentage of time for which pH remained > 4 during a 24-hour period, with secondary endpoints including the percentage of time for which pH remained > 4 during the first and last 12 hours of dosing and the mean pH over the 24-hour duration. We used pH > 4.0 as the threshold because maintaining a gastric pH >4.0 is widely recognized in acid suppression studies as the critical point below which acid-related damage can occur. Any instances where any data points were missing or incomplete have been resolved using standard imputation methods or excluding incomplete data points in the final analysis.

Sample size

This pilot study was performed on five randomized patients due to the complexity of the study methodology and stringent requirements for evaluability.

Ethical consideration

This study was conducted at a tertiary care center by a gastroenterologist in accordance with the ethical principles of the Declaration of Helsinki and Good Clinical Practice (GCP) guidelines. The study was approved by the Institutional Ethics Committee (IEC) (IEC/CE23011/270923) of Health1 Super Speciality Hospital, Ahmedabad (Protocol No: CE/23/011; Dated: 05/09/2023) before initiation and was subsequently registered on the Clinical Trial Registry of India (CTRI/2023/10/058381). All patients provided signed informed consent before enrollment. Patients were assigned to treatment groups using a computer-generated randomized schedule compiled by a third-party contract research organization (CRO), which had no involvement in the conduct of the study.

Statistical methods

End-point analyses were performed for the enrolled patients who received all doses in each of the five treatment periods. The mean percentage of time for which pH remained > 4.0 during the first 0-12-hour, 12-24 hour, and total 24-hour period were analyzed. A paired T-test was performed to compare the statistical difference between dexlansoprazole and other PPIs and placebo. A P-value less than 0.05 was considered significant.

## Results

**Figure 1:**
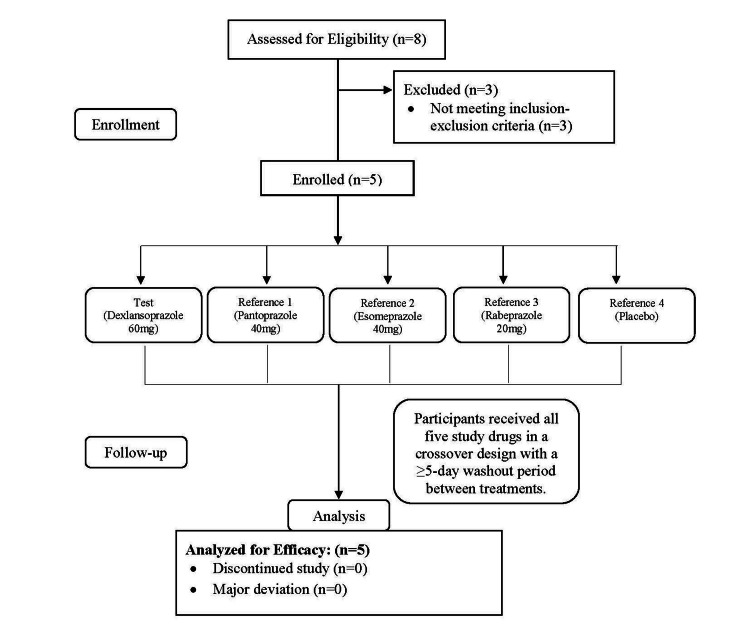
CONSORT diagram depicting the flow of patients in the study. CONSORT: Consolidated Standards of Reporting Trials.

A total of eight participants were assessed on the basis of their eligibility. Of these, five healthy volunteers were enrolled, each receiving all five study drugs in a crossover fashion after a washout period of at least five days (Figure 1).

**Figure 2:**
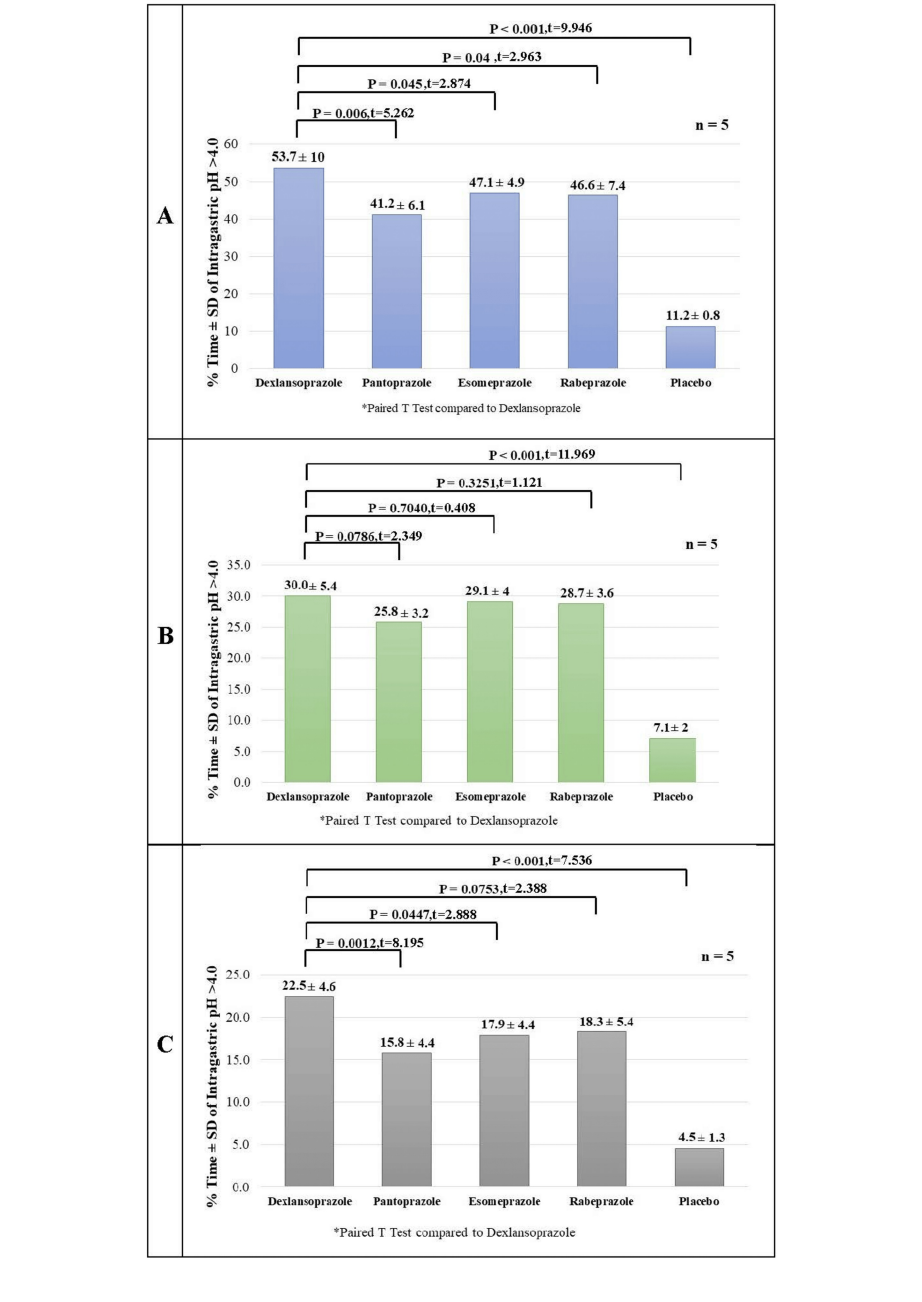
Mean percentage of time (%) for which intragastric pH remains > 4.0. Figure A depicts the mean percentage of time (%) for which intragastric pH remained > 4.0 during the 24-hour period. Figure B depicts the mean percentage of time (%) for which intragastric pH remained > 4.0 during the 0-12 hours period. Figure C depicts the mean percentage of time (%) for which intragastric pH remained > 4.0 during the 12-24 hours period.

The mean percentage of time for each treatment group for which the intragastric pH was greater than 4.0 during the 24-hour period is shown in Figure 2A. Figure 2B depicts the mean percentage of time for each treatment group in which the intragastric pH was > 4.0 during the first 0-12-hour period in which dexlansoprazole exhibited it for higher than four hours as compared to other PPIs and placebo; however, it was not statistically significant. Figure 2C illustrates the mean percentage of time for each treatment group in which the intragastric pH was > 4.0 during the last 12-24-hour period. This highlights the benefit of the dual-delayed release profile of dexlansoprazole over other PPIs.

**Figure 3:**
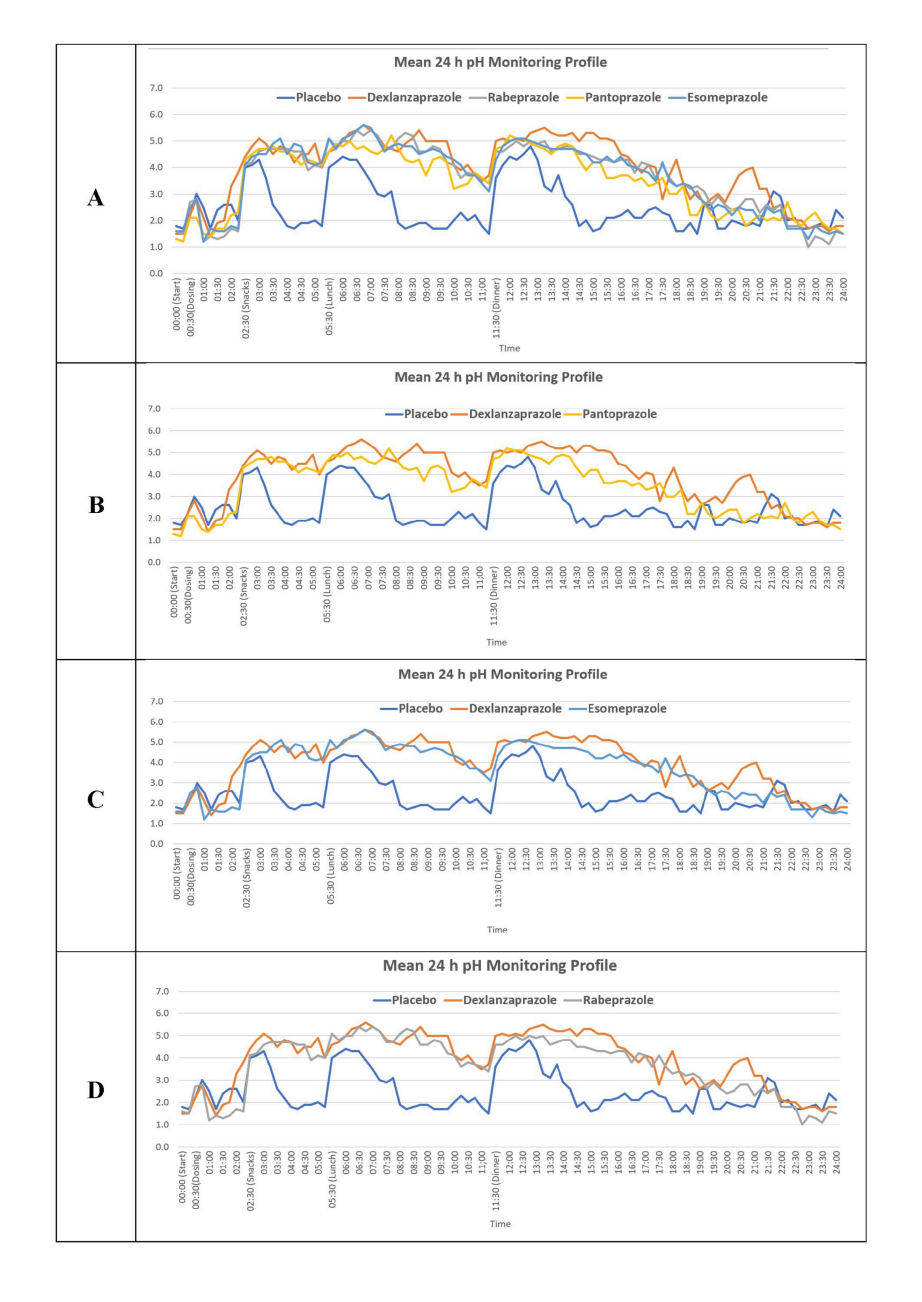
Mean 24-hour pH monitoring profile of dexlansoprazole, rabeprazole, pantoprazole, esomeprazole, and placebo. Figure A depicts the mean 24-hour pH monitoring profile of all treatment cohorts. Figure B depicts the mean 24-hour pH monitoring profile for dexlansoprazole and pantoprazole. Figure C depicts the mean 24-hour pH monitoring profile for dexlansoprazole and esomeprazole. Figure D depicts the mean 24-hour pH monitoring profile for dexlansoprazole and rabeprazole.

The mean pH values recorded at every 30-minute duration during the 24-hour pH recording are shown in Figure 3.

The mean pH over 24 hours (Table 1) for dexlansoprazole was found to be 3.98 in comparison with pantoprazole (3.48), esomeprazole (3.66), rabeprazole (3.66), and placebo (2.52), wherein the mean pH was found to be significantly greater in dexlansoprazole over pantoprazole and esomeprazole.

**Table 1:** Mean pH values recorded over 24-hour study duration as compared to dexlansoprazole. * Paired T-test compared to dexlansoprazole. SEM: standard error of the mean.

Proton pump inhibitors	Mean pH ± SEM	P-value (t-value)*
Dexlansoprazole	3.98 ± 0.11	NA
Pantoprazole	3.48 ± 0.12	0.0021 (7.071)
Esomeprazole	3.66 ± 0.05	0.0453 (2.874)
Rabeprazole	3.66 ± 0.05	0.0723 (2.426)
Placebo	2.52 ± 0.12	<0.001 (3.225)

Subsequently, a comparative analysis of the time for which pH was higher in dexlansoprazole with other PPIs was carried out in which dexlansoprazole demonstrated an increased effect of six hours and 24 minutes of higher intragastric pH than rabeprazole. Specifically, the pH of dexlansoprazole was elevated for 12 hours and 24 minutes, while rabeprazole exhibited a higher pH for six hours. Similarly, in comparison with pantoprazole, dexlansoprazole showcased a greater effect of eight hours of higher intragastric pH and it remained elevated for 12 hours and 18 minutes, whereas pantoprazole exhibited a higher pH for four hours and 18 minutes. In comparison with esomeprazole, dexlansoprazole displayed an elevated effect of five hours and 27 minutes of higher intragastric pH, wherein the pH was observed to be higher for 11 hours and 57 minutes, while esomeprazole exhibited a higher pH for six hours and 30 minutes. These findings highlight the varying durations and net effects of intragastric pH elevation associated with dexlansoprazole in comparison to rabeprazole, pantoprazole, and esomeprazole, providing valuable insights into the acid-suppressive capabilities of these PPIs.

## Discussion

The study results provide valuable insights into the gastric acid suppressive effects after a single dose of different PPIs. The four PPIs that were evaluated in the study showed at least 41.4% gastric acid suppression (pH > 4) in a 24-hour period. Dexlansoprazole (60 mg) provided an intragastric pH greater than 4.0 for a significantly greater duration (53.7%) of a 24-hour period as compared with pantoprazole, esomeprazole, and rabeprazole in healthy subjects. Dexlansoprazole also had a higher average intragastric pH than the other PPIs. Dexlansoprazole offers superior and highly significant acid suppression capability compared to placebo (11.2 ± 0.8) establishing its effectiveness over a 24-hour period.

Analysis of the pH duration during different intervals (0-12 hours and 12-24 hours) revealed notable trends. While dexlansoprazole demonstrated a longer duration of pH > 4 during both intervals compared to other PPIs and placebo, statistical significance was achieved only during the 12-24-hour period, particularly against pantoprazole and esomeprazole. For all efficacy endpoints, the results were observed to be statistically significant with dexlansoprazole compared with pantoprazole and esomeprazole in the 12-24-hour time frame thereby highlighting the dual-delayed release mechanism of dexlansoprazole. The different PPIs, pantoprazole, esomeprazole, and rabeprazole, are known to be effective in reducing acid suppression during the latter 12 hours of the day due to their insufficient absorption and the inherent limitation of single drug release kinetics. This limitation is often overcome by adjusting to a twice-daily dosing regimen, especially in refractory cases. In contrast, dexlansoprazole, harnessing its dual-delayed release mechanism, prolongs acid suppression over 24 hours. Hence, a once-daily dosing regimen is adequate for patients requiring extended acid suppression without taking multiple doses, making it a preferred option over the current PPIs.

The results of this study are in line with previous similar published studies. In a study conducted by Kukulka et al., dexlansoprazole was compared with esomeprazole wherein it demonstrated statistically significant differences in average pH with values observed to be 4.3 over 3.7 exhibited by esomeprazole 40 mg (p = 0.003). The proportion of mean percentage of time for which pH > 4 was observed to be 58% for dexlansoprazole compared to 48% for esomeprazole (p < 0.001) [31]. Additionally, in a study performed by Lee et al., the dual delayed-release formulation of dexlansoprazole demonstrated superior acid control compared to lansoprazole, with a prolonged duration of acid suppression throughout the 24-hour period [32]. From the results of these two studies, sustained acid inhibition is attributed to the unique pharmacokinetic profile of dexlansoprazole, providing an extended release of the active drug.

In another study aimed to compare the antisecretory activity of single doses of various PPIs (rabeprazole, lansoprazole, pantoprazole, omeprazole capsule, omeprazole multiple unit pellet system (MUPS) tablet, and placebo) in 18 healthy subjects, intragastric pH over 24 hours with rabeprazole was observed to be 3.4 while that with lansoprazole, pantoprazole, omeprazole capsule, omeprazole MUPS tablet, and placebo was observed to be 2.9, 2.2, 1.9, 1.8, and 1.3, respectively. Daytime and night-time pH values were also found to be 3.6 and 2.3 for rabeprazole, 3.3 and 2.1 for lansoprazole, 2.2 and 1.6 for pantoprazole, 1.8 and 1.6 for omeprazole capsule, and 2.1 and 1.5 for omeprazole MUPS tablet [33]. These data are in line with the findings of the current study.

Based on extensive clinical investigations involving a cohort of over 4,500 patients across seven distinct clinical trials, dexlansoprazole has demonstrated a commendable safety profile. Administered orally at dosages of 30 mg, 60 mg, and 90 mg, the drug exhibited infrequent adverse reactions, with instances predominantly of mild or moderate intensity. Reported adverse effects, including diarrhea (leading to discontinuation in 0.7% of cases), abdominal pain, headache, nausea, abdominal discomfort, flatulence, and constipation, were either comparable to or lower than those observed for the placebo or lansoprazole comparator [29,34,35].

Consistent with the behavior of other PPIs, dexlansoprazole led to a more than two-fold increase in gastrin levels during the initial three months of therapy, stabilizing thereafter without discernible clinical or morphological repercussions. The enduring efficacy of prolonged treatment in effectively managing reflux symptoms and enhancing the patient's quality of life is maintained throughout the duration of maintenance therapy, as reaffirmed by a one-year follow-up study [34,36].

Dexlansoprazole stands out as a remarkable therapeutic agent, distinguished by its ability to free patients from rigid mealtime constraints and specific drug administration schedules while maintaining therapeutic efficacy regardless of these temporal considerations [27]. Serving as the R-enantiomer of lansoprazole, dexlansoprazole constitutes over 80% of the bioavailable compound post-oral dosing, with a metabolic clearance rate that affords it a systemic exposure fivefold greater than the S-enantiomer of lansoprazole [27,30]. Notable improvements in the chemical structure and pharmaceutical formulation of dexlansoprazole have been implemented to enhance its bioavailability, metabolic profile, and efficacy in suppressing proton pump activity within gastric parietal cells [37].

Dexlansoprazole employs an innovative dual-release technology, releasing its active component in two distinct phases activated at different pH thresholds and temporal intervals. This results in dual peak plasma concentrations, yielding a total serum dexlansoprazole concentration three times than that of the left-handed enantiomer, with prolonged suppression of acid secretion compared to other available PPIs [4,29,37]. Approximately 25% of the total dosage is liberated within the proximal duodenal tract at pH 5.5, while the remaining 75% is released in the distal small intestine at pH 6.75, leading to two discrete peak drug concentrations in serum [27].

In a considerable number of GERD patients, conventional PPI therapy frequently proves to be inadequate in effectively managing its symptoms [38]. In this context, dexlansoprazole emerges as a promising alternative, offering enhanced efficacy and dosing flexibility. Transitioning from twice-daily esomeprazole to once-daily dexlansoprazole for maintenance therapy results in well-controlled symptoms and improved quality of life for most patients [32]. This improvement can be attributed to the pharmacokinetic properties of dexlansoprazole, which play a significant role in enhancing its effectiveness, as studies have indicated rapid achievement of therapeutic concentrations and sustained suppression of acid over a 24-hour period [8].

The main intention behind employing a crossover design was to eliminate physiological variations in gastric acid pH measurement, while randomization allowed for the prevention of selection bias. The main limitation of the current study is the small sample size, which can lower its statistical significance and generalizability potential; however, even with the limited sample size of this pilot study, statistically significant differences were observed. Moreover, to provide more reliable comparisons and show variability among various PPIs relevant for clinical application, bigger cohort research is necessary. To further evaluate and validate these results, additional investigations with larger cohorts are recommended. Additionally, while this crossover study was conducted at a single center, future studies involving multiple centers could provide a broader perspective and substantiate the role of dexlansoprazole in clinical settings.

The study contributes significant evidence to the comparative efficacy of various PPIs in modulating intragastric pH. Dexlansoprazole emerges as a potent acid suppressor with variable but generally favorable outcomes compared to other PPIs, highlighting its potential clinical advantages in managing acid-related disorders. These findings may inform clinical decision-making and treatment strategies for patients with acid-related gastrointestinal conditions. Further research is warranted to explore the long-term implications and clinical outcomes associated with these observed differences in intragastric pH modulation.

## Conclusions

The findings of this pilot study highlight the superiority of dexlansoprazole over other PPIs. Dexlansoprazole demonstrates dual-delayed release characteristics within the body, leading to sustained maintenance of pH levels > 4 for an extended duration over 24 hours. This unique attribute, coupled with its enantiomeric advantage derived from its dextrorotatory configuration as an enantiomer of lansoprazole, enhances its capacity to suppress gastric acid production, distinguishing it from its counterparts within the PPI class. Its prolonged 24-hour acid-suppression profile is particularly noteworthy, providing sustained relief and benefiting individuals, particularly those afflicted by nocturnal reflux and resultant sleep disturbances. Moreover, its favorable safety profile, characterized by infrequent and mild adverse effects, makes dexlansoprazole suitable for long-term therapeutic use. In light of these attributes, dexlansoprazole also offers the advantage of flexible meal timing, a feature that promotes patient adherence and cooperation with daily clinical routines. The findings of this pilot study suggest that dexlansoprazole demonstrates a dual-delayed release mechanism and superior pH control, which allows for its administration once daily, thereby enhancing patient convenience in real-world clinical practice. However, a larger, adequately powered study is necessary to confirm and expand upon these observations and to further evaluate the long-term efficacy and safety of dexlansoprazole.
